# Retraction: Enantioselective synthesis of α-tetrasubstituted (3-indolizinyl) (diaryl)methanamines *via* chiral phosphoric acid catalysis

**DOI:** 10.1039/d4ra90001d

**Published:** 2024-01-08

**Authors:** Jialing Zhong, Rihuang Pan, Xufeng Lin

**Affiliations:** a Department of Chemistry, Zhejiang University Hangzhou 310027 P. R. China lxfok@zju.edu.cn

## Abstract

Retraction of ‘Enantioselective synthesis of α-tetrasubstituted (3-indolizinyl) (diaryl)methanamines *via* chiral phosphoric acid catalysis’ by Jialing Zhong *et al.*, *RSC Adv.*, 2022, **12**, 20499–20506, https://doi.org/10.1039/D2RA03750E.

We, the named authors, hereby wholly retract this *RSC Advances* article. In this article, an incorrect version of molecular structure for product **3p** based on its X-ray structure in Table 2 was included. Thus, the molecular structure and compound name for products **3a–q** and **4** in Tables 1–2 and Schemes 1–3 should be revised accordingly. In addition, ‘3-indolizinyl’ should be corrected to ‘1-indolizinyl’ in the title, abstract and the main text of the article. All the reported yields, data, or spectral information provided in the article remain valid.

Having consulted with an independent expert, the Royal Society of Chemistry has determined that any changes made to the paper to correct this would be significant, and therefore the best course of action is retraction and republication of the article with the correct molecular structure of products. The Royal Society of Chemistry is happy that the overall conclusions of the paper are not affected by this error, and therefore that republication of the work with the correct molecular structure of products is appropriate. The republished article was peer reviewed and can be found at https://doi.org/10.1039/D3RA07636A.

We, the authors, brought this matter to the attention of the Royal Society of Chemistry ourselves, and are happy with the decision to retract and republish this article.

Signed: Jialing Zhong, Rihuang Pan and Xufeng Lin, 23rd November 2023.

Retraction endorsed by Laura Fisher, Executive Editor, *RSC Advances*.

## Supplementary Material

